# Supplementary material for: ERK inhibits Capicua repressor function via multisite phosphorylation
Source: J Cell Sci. 2026 Mar 30;139(6):jcs264327. doi: 10.1242/jcs.264327 (PMC13086492; doi:10.1242/jcs.264327)
Supplement: Supplementary information [file joces-139-264327-s1.pdf]

site	run1	run2	run3	run4	total	Cic(1-15)A	Cic6A	Cic20A
S41	0	1	0	0	1			
S49	0	1	0	0	1			
T63	0	1	1	1	3			yes
T296	1	1	1	1	4	site 1		yes
S299	0	1	0	0	1			
T430	0	1	1	1	3			yes
S459	0	1	0	0	1			
T480	1	1	0	1	3	site 2		yes
S501	0	0	1	0	1			
T573	1	1	0	1	3	site 3		yes
T701	1	1	1	1	4	site 4		yes
S716	0	0	0	1	1			
S736	0	1	1	1	3			yes
S773	0	0	0	1	1			
T781	1	1	1	1	4	site 5		yes
T786	1	0	0	1	2	site 6		yes
S809	0	1	1	1	3			yes
S881	0	0	0	1	1			
T883	0	0	0	1	1			
S895	0	1	1	1	3			yes
S896	0	0	0	1	1			
T910	0	1	1	0	2		yes	yes
S914	1	1	1	1	4	site 7	yes	yes
S943	1	0	0	0	1	site 8		
S950	1	1	0	0	2	site 9	yes	yes
S1010	1	1	0	0	2	site 10	yes	yes
S1014	1	1	1	1	4	site 11	yes	yes
T1016	0	0	1	0	1			
S1020	1	1	1	1	4	site 12	yes	yes
T1059	0	1	1	0	2	no (C2 domain)		
T1231	1	1	1	1	4	site 13		yes
S1251	1	1	1	1	4	site 14		yes
T1287	0	0	0	1	1			
S1291	0	1	1	1	3			yes
S1310	1	0	0	0	1	site 15		

Fig. S1. ERK-mediated Cic S/T phosphorylation sites identified by mass spectrometry (biological replicates 1-4) and sites chosen for mutagenesis. The three rightmost columns show the sites mutated to alanine in the corresponding Cic variants: $Cic^{(1-15)A}$ was based on the first biological replicate (run 1), and Cic^{6A} and Cic^{20A} were based on all four runs. Cic^{20A} includes all sites identified in at least two runs, except T1059 located in the C2 domain, which is the binding site for ERK. Cic^{6A} is a subset of Cic^{20A}. Output from the Ascore algorithm for phosphosite identification is shown in Dataset S1.

Journal of Cell Science • Supplementary information

Capicua, isoform A (NCBI ID: NP_524992.1)

MNAFQDFELGAKLYLQCLLSLSSSRSATPSYTSPVNHAGASPLNAIAHSPVNVSATHRQNFFTPIANQSQQQQQQQPVAV (1–80)

PLDSKWKTTPSPVLYNANNNSSNNNTSSSNNNNNSNWEVGSNSNTHVAATAAATSTVGAQPLPPQTTPVSLVMHAPPPQQ (81–160)

QPLQQQHHHHQPPPPPPASLPAPSAPPTSGSSSSHNSVGHATSVIRISSSQQQHQQQQQHQQQAHPHVVVSGGQTFHPVI (161–240)

VDATQLSVPLPPTTVSFHQPNTPTSTAASVASMSQDKMLAKNGYNAPWFKLLPHMTPMSKASPAPVTPTLTTSASSYNVV (241–320)

MMQQQQQHQQLQQQQQLQQQQQSPPQMPLNHNNNHLIVSAPLSSPGKPLNCSMNDAKVAAAAAAAAVANQRQKQQQEEPD (321–400)

DQLDDDVFETTTPGISANSKKQTAAMRLPTHNSNIRKLEECHDDGAAGAPATSAAKRRSQSLSALQQQQQQQQQAGAAGT (401–480)

AAGQPANKKIRRPMNAFMIFSKKHRKMVHKKHPNQDNRTVSKILGEWWYALKPEQKAQYHELASSVKDAHFKLHPEWKWC (481–560)

HMG box

SKDRRKSSTSTATPGGKASGAAGTGDAKQRLVSVDGSDSLEHDMCPSTPGGSGSCGGQGISSDLQGDIIPLTIDNYNSTC (561–640)

DEAPTTISMKGNGNGKLMKNELPSDEDEQMLVVEEEQQQQTVKKIDLHCRERVNDSDMDDTPFDYRKQQPEANQRSAEEH (641–720)

STSGANGQAINAPPLSGGEREITLKPKAIKAHPVLESNMLPYTQMSIYTQYTSPKNPIGVTPFQPTGGAFKSMPISPKGS (721–800)

GGKPEDAGSLQAHIKQEDIKQEPPSPYKLNNGSGSASGGGVVSAPPPNSGSVGAIFNFNVPTATALSQKQFHYPMHHPHR (801–880)

SPTDLRAAHQACVPSSPAGMGLGHAANIATPPASAPAQIMGGGPASQKMFFAMTHPYTLLQRSHQPGTPSLEHLQLDAFA (881–960)

6A 6A

PGGYTLRNHNGLSSLPPPVSAQPTMLLHGYPPSHGVEPPARSPSYKSMPSTPKSATYLMSAPPERGMDGGMSGCASAAAS (961–1040)

6A 6A

GGDESDIDADGQQFILAPTPAQLGRAPLQRRKNLSQSKSESNVSFGANLGASNGQHISRKLHSPTMMESSSPIIGHVNSS (1041–1120)

C2 domain

NLSSALPTPTSSTTTPNSDEQLPLTPTTSSSNSNLNQQQPKSPMKGAPGSTAAALKKKNDEMNNSVLKQVDFEKKYKALP (1121–1200)

QFQPEDCQSPSAIAVPSSPRVYGTNYRKKNTAPPPVQKLMCEDDSIDEPASAPPTTTQRFFGPDFNNELKELESSDQTGR (1201–1280)

SPRTPKTPLQSARSDASEKGHRKVLETRRSLVLQLFAEHGNFPTAQATMAFQSKHSDVFPRKQDLQLKIREVRQKLLGQA (1281–1360)

C1 domain

SCTPHSAGPNTPSDSNSSSTTLSASSTSLNMQTTSAADVFQYY (1361–1403)

Journal of Cell Science • Supplementary information

Fig. S2. Annotated sequence of the short isoform of Capicua (isoform A) used in this study. S/T phosphorylation sites mutated in Cic^{20A} are shown in red. Green boxes labeled "6A" show putative Ago/FBXW7 phosphodegrons and include the sites mutated in Cic^{6A}. T1059 (blue) was identified as being phosphorylated but was not mutated due to its location in the C2 domain that interacts with ERK.

Journal of Cell Science • Supplementary information

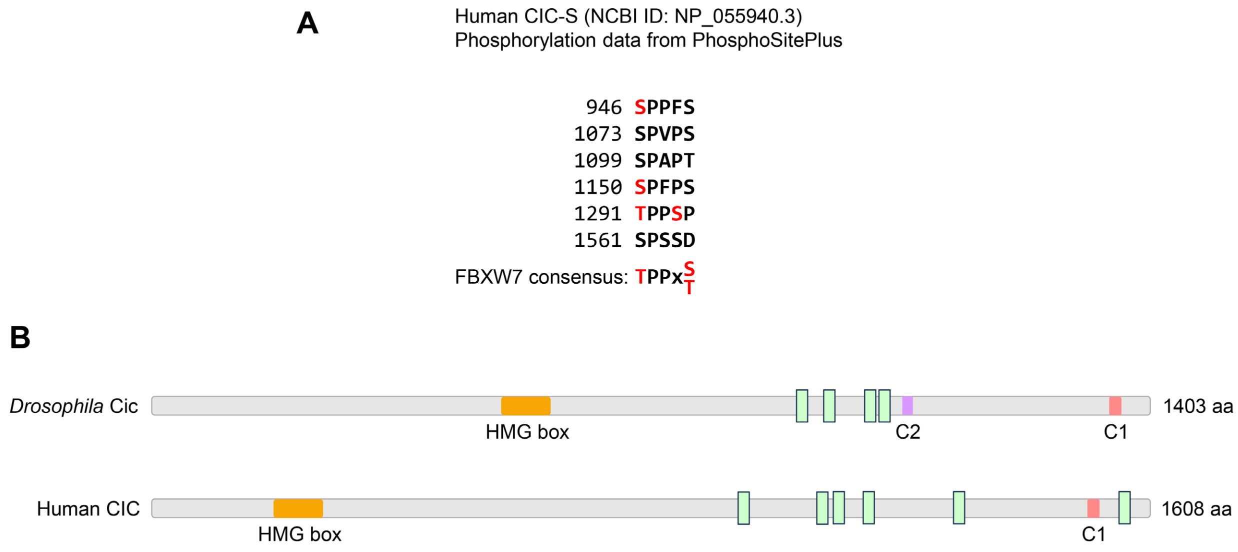

Fig. S3. Predicted FBXW7 phosphodegrons in human CIC. (A) Potential FBXW7 phosphodegrons in the human CIC-S protein. Phosphorylated residues are shown in red (data from PhosphoSitePlus). (B) Relative locations of predicted phosphodegrons in *Drosophila* Cic and human CIC proteins. Phosphodegrons are indicated as light green boxes.

Table S1. Cic phosphorylation sites identified by mass spectrometry.

Available for download at
https://journals.biologists.com/jcs/article-lookup/doi/10.1242/jcs.264327#supplementary-data

Journal of Cell Science • Supplementary information
